# Metabolomics of COPD Pulmonary Rehabilitation Outcomes via Exhaled Breath Condensate

**DOI:** 10.3390/cells11030344

**Published:** 2022-01-20

**Authors:** Mauro Maniscalco, Debora Paris, Paola Cuomo, Salvatore Fuschillo, Pasquale Ambrosino, Annabella Tramice, Letizia Palomba, Andrea Motta

**Affiliations:** 1 Pulmonary Rehabilitation Unit of Telese Terme Institute, Istituti Clinici Scientifici Maugeri IRCCS, 82037 Telese Terme, Italy; salvatore.fuschillo@icsmaugeri.it; 2 Institute of Biomolecular Chemistry, National Research Council, 80078 Pozzuoli, Italy; debora.paris@icb.cnr.it (D.P.); pao.cuomo@gmail.com (P.C.); annabella.tramice@icb.cnr.it (A.T.); 3 Cardiac Rehabilitation Unit of Telese Terme Institute, Istituti Clinici Scientifici Maugeri IRCCS, 82037 Telese Terme, Italy; pasquale.ambrosino@icsmaugeri.it; 4 Department of Biomolecular Sciences, University of Urbino “Carlo Bo”, 61029 Urbino, Italy; letizia.palomba@uniurb.it

**Keywords:** chronic obstructive pulmonary disease, biomarkers, metabolomics, NMR, outcomes, personalized therapies, rehabilitation, exercise, disability

## Abstract

Chronic obstructive pulmonary disease (COPD) is characterized by different phenotypes and clinical presentations. Therefore, a single strategy of pulmonary rehabilitation (PR) does not always yield the expected clinical outcomes as some individuals respond excellently, others discreetly, or do not respond at all. Fifty consecutive COPD patients were enrolled. Of them, 35 starting a 5-week PR program were sampled at admission (T_0_), after 2 (T_2W_) and 5 (T_5W_) weeks, while 15 controls not yet on PR were tested at T_0_ and T_5W_. Nuclear magnetic resonance (NMR) profiling of exhaled breath condensate (EBC) and multivariate statistical analysis were applied to investigate the relationship between biomarkers and clinical parameters. The model including the three classes correctly located T_2W_ between T_0_ and T_5W_, but 38.71% of samples partially overlapped with T_0_ and 32.26% with T_5W_, suggesting that for some patients PR is already beneficial at T_2W_ (32.26% overlapping with T_5W_), while for others (38.71% overlapping with T_0_) more time is required. Rehabilitated patients presented several altered biomarkers. In particular, methanol from T_0_ to T_5W_ decreased in parallel with dyspnea and fatigue, while the walk distance increased. Methanol could be ascribed to lung inflammation. We demonstrated that the metabolic COPD phenotype clearly evolves during PR, with a strict relationship between clinical and molecular parameters. Methanol, correlating with clinical parameters, represents a useful biomarker for monitoring personalized outcomes and establishing more targeted protocols.

## 1. Introduction

Currently, chronic obstructive pulmonary disease (COPD) is the third leading cause of death worldwide [1]. In recent years, given the rising social and economic costs, COPD prevention and treatment have received growing attention [2], and pulmonary rehabilitation (PR), although underused [3], has become essential for COPD management. PR is a multidisciplinary approach based on exercise training and pharmacological and non-pharmacological (e.g., physical, psychological, educational, and nutritional) interventions [4]. Expected PR outcomes are improvement in dyspnea, quality of life, exercise tolerance, and a reduction in hospitalization [4]. However, COPD remains a disease with a wide spectrum of clinical presentations, with different phenotypes even in patients with a comparable degree of airflow limitation [5]. Therefore, the individual response to PR is highly variable and sometimes unpredictable [6], because “there is currently no standardized way to assess which model would best suit which patient (and vice versa)” [3]. As a consequence, COPD management would highly benefit from unbiased molecular biomarkers of PR outcomes.

Since metabolomics can identify and quantify the small molecules produced during biological processes, it has become an essential tool for phenotyping respiratory diseases [7]. Furthermore, metabolomics can map metabolic pathways altered by the diseases’ pathophysiology, thus having a key role in discovering novel biomarkers and monitoring treatment response [8]. Recently, nuclear magnetic resonance (NMR)-based metabolomics of exhaled breath condensate (EBC) has been proposed as a rapid and non-invasive tool for investigating airway diseases [9,10]. EBC is a natural matrix that can be easily, non-invasively, and repeatedly obtained by cooling exhaled air from spontaneous tidal breathing. Because of its origin, it likely reflects airway inflammation [11], and its study by NMR-based metabolomics has become a standardized method with practical clinical applications in chronic respiratory diseases [12].

We postulated that EBC metabolomics and clinics could usefully interact for phenotyping rehabilitating COPD patients, carefully describing physiological changes and detecting possible biomarkers that could define PR outcomes and personal rehabilitation treatment. By using NMR-based metabolomics of EBC, we here identified dysregulated biomarkers of clinical and functional response in a cohort of COPD patients undergoing in-hospital PR.

## 2. Materials and Methods

### 2.1. Patients

For this single-center prospective controlled study, consecutive COPD patients referring to the Pulmonary Rehabilitation Unit of Istituti Clinici Scientifici Maugeri Spa SB, IRCCS of Telese Terme (Benevento), Italy, were screened for eligibility. COPD patients undergoing PR were enrolled as cases, while those not yet on PR served as controls.

The only major inclusion criterion was the presence of an objectively confirmed diagnosis of COPD, according to Global Obstructive Lung Disease (GOLD) guidelines [6]. Exclusion criteria were a history of asthma or any respiratory disease other than COPD; a diagnosis of alpha-1 antitrypsin deficiency; any previous lung surgery; a history of any immune-mediated chronic inflammatory disease; history of cardiovascular diseases (acute myocardial infarction, congestive heart failure, stroke); current malignant disease or a diagnosis of malignancy in the 2 years prior to the first visit; an exacerbation of COPD; hospitalization or change in drug treatment during the 4 weeks prior to the first visit; inability to walk; any blood transfusion 4 weeks prior to the first visit; suspicion of alcohol or drug abuse, or non-complete adherence to exercise training and time schedule, as well as motivation.

The study was conducted in accordance with the Declaration of Helsinki of the World Medical Association. The study was approved by the Institutional Review Board of Istituto Nazionale Tumori, Fondazione Pascale, Naples, Italy, with reference number ICS 2/17, and all patients provided written informed consent to use their de-identified data for future research.

### 2.2. Study Procedures

After informed consent signature, a detailed medical history was recorded for each patient. For COPD patients starting the 5-week PR program, all study procedures were performed at admission (T_0_) and repeated after 2 (T_2W_) and 5 (T_5W_) weeks. For control subjects (COPD patients not on PR), only two evaluation time-points were considered (T_0_ and T_5W_).

### 2.3. Pulmonary Function Tests (PFTs)

For each patient, post-bronchodilator forced expiratory volume in the first second (FEV_1_) and forced vital capacity (FVC) were measured using standardized spirometry. Their ratio (FEV_1_/FVC) was also calculated. Lung volumes, flow rates, and single breath carbon monoxide diffusing capacity (DLCO) were determined using an automated equipment (Vyasis, Milan, Italy) in agreement with standardized procedures [13]. A comprehensive assessment of symptoms and risk of exacerbations was performed at admission in the frame of the refined ABCD risk assessment tool [14].

### 2.4. 6-Minute-Walk Test (6MWT)

The 6MWT was performed at each time-point according to American Thoracic Society (ATS) recommendations [15]. The test was performed along an air-conditioned hospital corridor 15 m long. The walk was symptom-limited, allowing patients to pause if necessary, and continue after they had rested. The covered distance was expressed in meters. During the test, the oxygen saturation was continuously monitored by a pulse oxymeter via a finger electrode. Patients were asked to rate their symptoms at the beginning and at the end of the test using the modified Borg CR10 scale [16]. The Borg CRD10 scale represents a valid and widespread tool for measuring the intensity of perceived dyspnea and physical exertion in a numerical range between 0 and 10. Before the 6MWT, each patient was instructed to transform the severity of perceived dyspnea and muscle fatigue into a numerical value knowing that 0 expresses the absence and 10 the maximum of perceived severity of both dyspnea and fatigue.

### 2.5. Pulmonary Rehabilitation

Patients underwent a 5-week PR program with daily sessions (6 sessions/week). The program consisted of 30 sessions following the official ATS/ERS statement [17], including physical exercise training, dietary and psychosocial counseling. Physical exercise training included exercises to strengthen groups of muscle in the upper and lower extremities, treadmill walking and stationary cycling. Lower limb strengthening exercises were performed using body weight (squats, sit-to-stands, and step-ups), and fixed weights (leg extension and leg press) at a load that could be supported for 8–10 repetitions before muscle exhaustion. A similar overload stimulus was considered for upper extremities, with both free and fixed weights (pull downs and chest press) being used. Resistances were increased once participants were able to complete three sets of 8–10 repetitions in two consecutive training sessions. Arm ergometry was performed for 10 min per session at an intensity of 3–4 on the Rating of Perceived Exertion (RPE) 0–10 scale [18]. Initial treadmill walking duration was 15 min and was progressed to 30 min within the first 2 weeks from admission, aiming at an RPE score of 3–4. Similarly, lower limb cycling intensity was set at an intensity aimed at scoring dyspnea or perceived exertion from 3 to 4 on the modified 0–10 category-ratio scale [19]. All patients also underwent flexibility and stretching exercises. Participation was supervised and monitored by physiotherapists.

### 2.6. EBC Collection

All subjects were asked to refrain from food intake for 8 h, and from alcoholic drinks for 18 h, which they confirmed before sample collection. EBC was collected in a random order and in the same room with a TURBO-DECCS condenser (Medivac, Pilastrello, Parma, Italy, www.medivac.it, accessed on 30 November 2021) set at −5.0 ± 1.0 °C as reported [20]. We obtained, on average, 2.0 ± 0.3 mL (mean ± SD) of EBC from each subject. Salivary contamination of the samples was tested by measuring their α-amylase activity, and using 1D-NMR spectra, in which contaminated spectra present signals from carbohydrates (absent in EBC spectra). The room temperature remained constant (24 ± 1.0 °C) throughout the sampling period. Possible air contaminants in the collecting room were monitored with a dedicated sampling pump for air monitoring (Zambelli EGO PLUS TT; Zambelli, Milan, Italy), working at a flow rate of 8 min per liter and tidal volume (500 mL) into the condenser, so as to simulate human breath. The pump was connected to the condenser outlet for 15 min, and special filters (3M Particulate Filters P100; 3M Italia, Milan, Italy; tested against particles approximately 0.3 μm in size) for respiratory protection were applied to the one-way valve of the mouthpiece condenser used for the whole set of experiments. NMR spectra of condensed room air from the collecting device were devoid of signals, confirming the absence of air pollutants (data not shown).

To reduce the risk of contamination by inhaling hospital air, subjects were sampled after a 30 min rest in the greenhouse of the Department of Respiratory Medicine, which was shown to be contaminant free as described above for the collecting room.

### 2.7. NMR Sample Preparation and Spectra Acquisition

EBC samples were rapidly defrosted. To provide a field frequency lock, 70 μL of a ^2^H_2_O solution (deuterated water, containing 0.1 mmol per liter 3-(Trimethylsilyl)propionic-2,2,3,3-d4 acid sodium salt (TSP) as a chemical shift reference for ^1^H spectra and sodium azide at 3 mmol per liter as a bacteriostatic agent) was added to 630 μL of EBC, reaching 700 μL of total volume. NMR spectra were recorded on a Bruker Avance III 600 MHz spectrometer (BrukerBioSpin GmbH, Rheinstetten, Germany) equipped with a CryoProbe and an automatic and cooled sample changer of 24 positions controlled by the software ICON-NMR program suite (TOPSPIN 3.60 version, BrukerBioSpin GmbH, Rheinstetten, Germany). Sample temperature was kept constant at 300 K (27 °C). 1D spectra including water suppression with excitation sculpting sequence [21], together with homo- and heteronuclear 2D experiments (^1^H-^1^H clean TOCSY and ^1^H-^13^C HSQC) were acquired as previously described [9,10].

### 2.8. Power Analysis

For projection methods, the power of the analysis cannot be evaluated by standardized methods. In metabolomics studies, a priori power analysis is not possible because biomarkers and their concentration variations are not known before analysis [22]. For an estimation, the 1 − α and 1 − β parameters were varied from 95% to 99.9% and from 80% to 99.9%, respectively. Using the accuracy percentages obtained in our validation tests (see Results and [22]) for 1 − α = 95% and 1 − β = 80%, we derived 22 ± 3 COPD patients for all classes, while for 1 − α = 1 − β = 99.9% we obtained 25 ± 2 patients. To account for possible drop-outs or protocol adherence issues, we screened 100 COPD patients for eligibility, with the final patients exceeding the numbers obtained from the backward analysis. Normally, 1 − α = 95% and 1 − β = 80%, while 99.9% represents an extreme condition.

### 2.9. Multivariate Data Analysis

EBC proton spectra ranging from 8.60 to 0.60 ppm were automatically binned into 400 integrals of 0.02 ppm each using the AMIX 3.9.15 software package (Bruker Biospin GmbH, Rheinstetten, Germany). The residual water resonance region (5.10–4.60 ppm) was excluded, and each integrated region was normalized to the total spectrum area to avoid possible dilution effects on the signals. The obtained NMR data format, expressed by a matrix (X matrix), was then imported into the software package Soft Independent Modeling of Class Analogy P version 14 (SIMCA-P+14) (Umetrics, Umeå, Sweden) where Principal Components Analysis (PCA) and Orthogonal Projections to Latent Structures Discriminant Analysis (OPLS-DA) were performed, after unit variance (UV) scaling. Initially, PCA was used to reduce data dimensionality and to explore possible trends and outliers. Once class homogeneity was assessed for each group, supervised OPLS-DA was applied to emphasize categories’ discrimination, where dummy variables were assigned to define class belonging (Y matrix). Supervised regressions were conducted comparing EBC groups at T_0_, T_2W_, and T_5W_ to generate predictive models that better relate metabolites variation to PR stages. Moreover, spectroscopic data were integrated with physical parameters like the 6MWT, fatigue, and dyspnea experienced at each time point to monitor the global PR effects on patients. For this, we applied OPLS searching for latent variables that maximize correlations between NMR and clinical parameters (treated as Y-variables). Each model quality was evaluated by using the goodness-of-fit parameter (R^2^) and the goodness-of-prediction parameter (Q^2^) [23] together with an internal iterative 7-round cross-validation and permutation test (800 repeats) and ANalysis Of VAriance testing of Cross-Validated predictive residuals (CV-ANOVA). To quantify the discriminatory metabolites, we selected the bins containing non-overlapping NMR signals, and used OriginPro 9.1 software package (OriginLab Corporation, Northampton, MA, USA) for the analysis. Statistical significance for selected metabolites was determined by parametric (ANOVA with Bonferroni correction) or non-parametric (Mann-Whitney U) tests according to the results of normality test performed on data to evaluate each distribution (Shapiro-Wilk, Kolgomorov-Smirnov test). *p* < 0.05 was considered as statistically significant. Finally, by combining clinical test values and selected bin integrals of significant metabolites, a correlation map with hierarchical clustering was also generated with the R software (www.R-project.org/, accessed on 30 November, 2021). The Euclidean distance was considered for the metrics, and the centroid method for clustering criterion.

Statistical analysis was performed with Prism 8.4.3 software package (GraphPad Software Inc, San Diego, CA, USA). Continuous data were expressed as mean ± standard deviation. Unpaired *t*-test was used for comparisons between rehabilitated COPD and controls. Paired *t*-test was used to evaluate differences between COPD before and after PR.

## 3. Results

### 3.1. Patients

**Figure 1:**
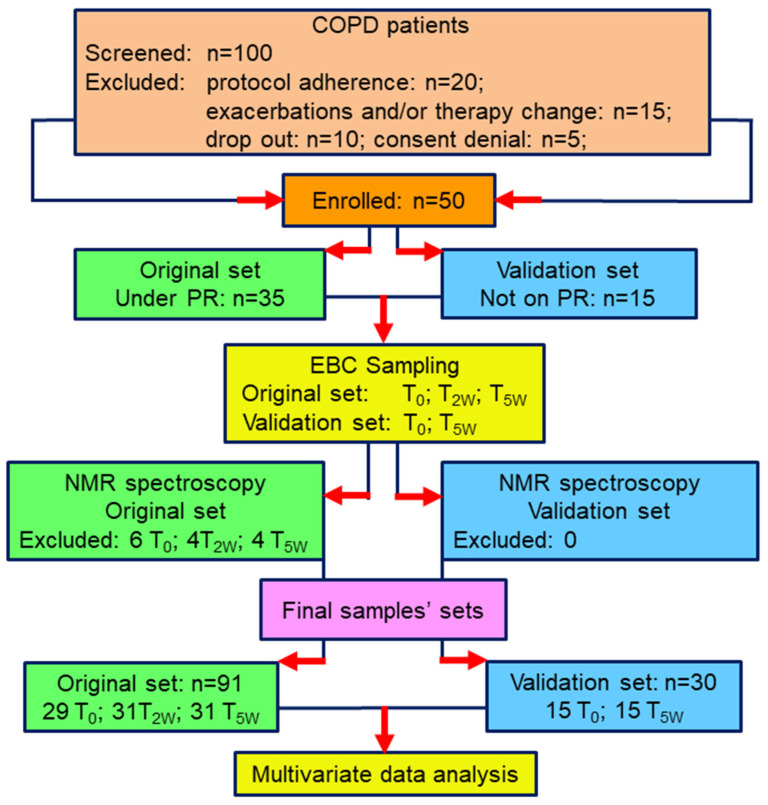
Schematic diagram illustrating the overall study design.

The study design is presented in Figure 1. We screened 100 consecutive COPD patients for eligibility. Twenty of them were excluded for protocol adherence, 15 because of exacerbations and/or change in the therapy during the study, while five refused to sign the informed consent. Ten patients dropped out before completion because of exacerbation and asked for removal of their data. Thus, 50 patients were enrolled: 35 starting a 5-week PR program, and 15 matched patients not on PR as controls. Except for six samples from T_0_, 4 from T_2W_, and 4 from T_5W_, which presented saliva contamination and/or NMR spectral distortion, the 91 final samples (29 T_0_, 31 T_2W_, and 31 T_5W_) were analyzed. The control group was sampled at T_0_ and T_5W_, for a total of 30 samples from all 15 patients. Three out of 35 cases and 1 out of 15 controls were Group C (high risk, less symptoms), while 32 out of 35 patients and 14 out of 15 controls were Group D (high risk, more symptoms) according to the refined ABCD risk assessment tool [15].

Table 1 reports the main demographic and clinical data of enrolled patients. For COPD patients under PR, major variations were observed at T_5W_ for 6MWT, fatigue, and dyspnea (column *p* value 1–2, *p* < 0.001). As expected, the T_0_ and T_5W_ corresponding parameters of the 15 controls were very similar, and with those at T_0_ of the PR set, since they were not on PR and were stable during the 5-week control period. Comparison between T_5W_ parameters for PR and controls presented significant values for clinical characteristics affected by PR (column *p* value 2–4), as the groups represent patients under PR (column 2) and controls not yet on PR (column 4).

### 3.2. NMR Profiling of EBC

**Figure 2:**
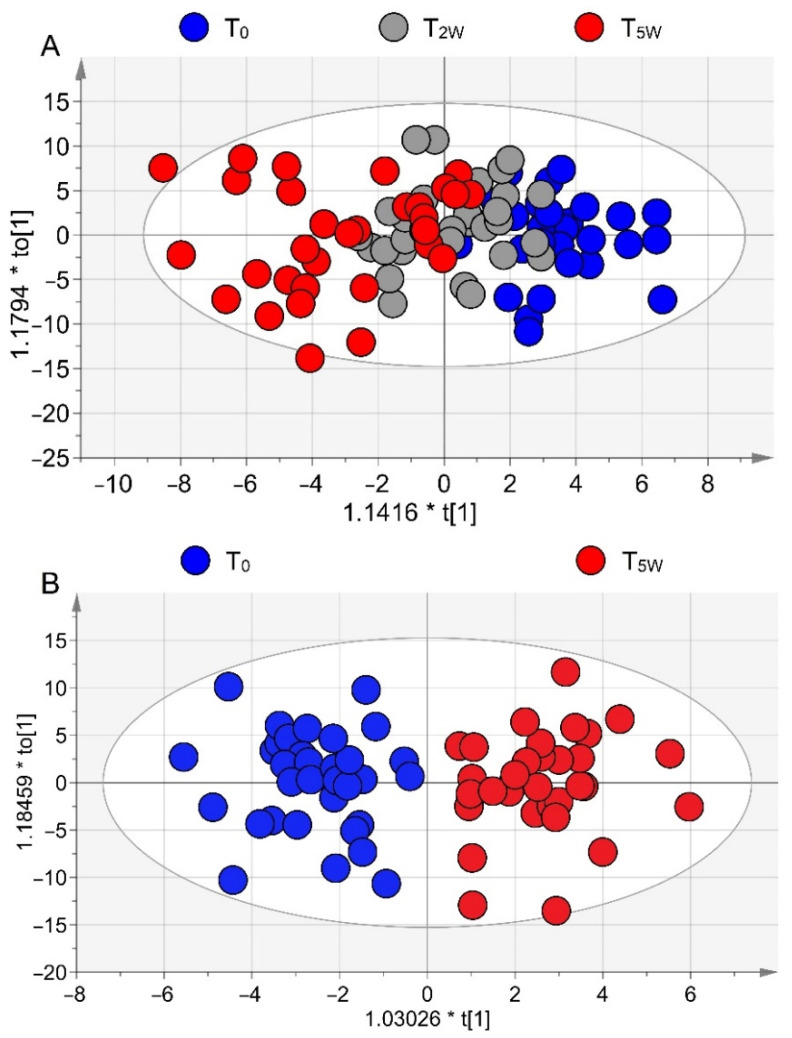
Orthogonal Projections to Latent Structures Discriminant Analysis (OPLS-DA) of EBC samples collected from COPD patients during the PR cycle. (**A**) Scores plot showing the degree of separation of the full model (T_0_, blue circles; T_2W_, gray circles; and T_5W_, red circles). (**B**) Scores plots showing the degree of separation of the model between T_0_ (blue circles) and T_5W_ (red circles) patients. The labels t[1] and t[2] along the axes represent the scores (the first two partial least-squares components) of the model, which are sufficient to build a satisfactory classification model.

EBC samples from patients under PR collected at T_0_, T_2W_, and T_5W_ were profiled via NMR-based metabolomics. After spectra acquisition, we applied unsupervised PCA (i.e., no prior knowledge is used in the calculations) to exclude class inhomogeneity and outliers. Supervised OPLS-DA of the three classes yielded the model (R^2^ = 0.69, Q^2^ = 0.49, and CV-ANOVA *p* = 0.010) depicted in the scores plot of Figure 2A. It shows a clear class evolution along t[1], with T_0_ (blue circles) and T_5W_ (red circles) well separated, but T_2W_ samples (gray circles) are equally distributed around the origin of the predictive component, with a partial overlap with the T_0_ group at positive t[1] and with the T_5W_ samples at negative t[1]. Such graphical overlap originates from “molecular overlap” that reflects similar pathophysiological states [24].

This suggests that PR does not progress linearly and homogeneously for all patients, and that its dynamics follow a variable induction period typical for each patient. Indeed, as shown in the misclassification Table 2 (Fisher’s *p* = 6.7 × 10^−7^), only 29.03% of T_2W_ samples resulted correctly classified (9/31), while the remaining 70.97% were partly categorized in T_0_ (12/31) and in T_5W_ (10/31) classes.

This implies that for some patients, PR is already effective at T_2W_ (gray circles overlapping with red circles, 32.26% of patients), while for others (gray circles overlapping with blue circles, 38.71% of patients) more time is required to become beneficial. Interestingly, a statistically significant concentration variation was found for methanol, with the highest level at T_0_ and a progressive decrease at T_2W_ (*p* < 0.05, indicated with *) and T_5W_ (*p* < 0.001, **) (Figure S1A).

Contiguous two-class OPLS-DA models (T_0_–T_2W_ and T_2W_–T_5W_) presented no statistical significance. The only significant model was T_0_–T_5W_, with high-quality parameters (R^2^ = 0.83, Q^2^ = 0.53; CV-ANOVA *p* = 0.0002). The scores plot (Figure 2B) indicated that EBCs collected at T_0_ and T_5W_ are clearly different, signifying that the metabolic phenotype (“metabotype”) changes after 5 weeks (T_5W_). From the corresponding loadings plot (not shown), after 5 weeks, rehabilitated COPD patients presented a reduction in acetate, methanol, 3-hydroxyisovalerate, isobutyrate, and acetone levels together with an increase of isopropanol, 1-propanol, lactate, and fatty acids levels (Table S1). Statistically significant metabolites are reported as box-and-whiskers plots in Figures S1 (panels B–D) and S2 (panels A–D).

**Figure 3:**
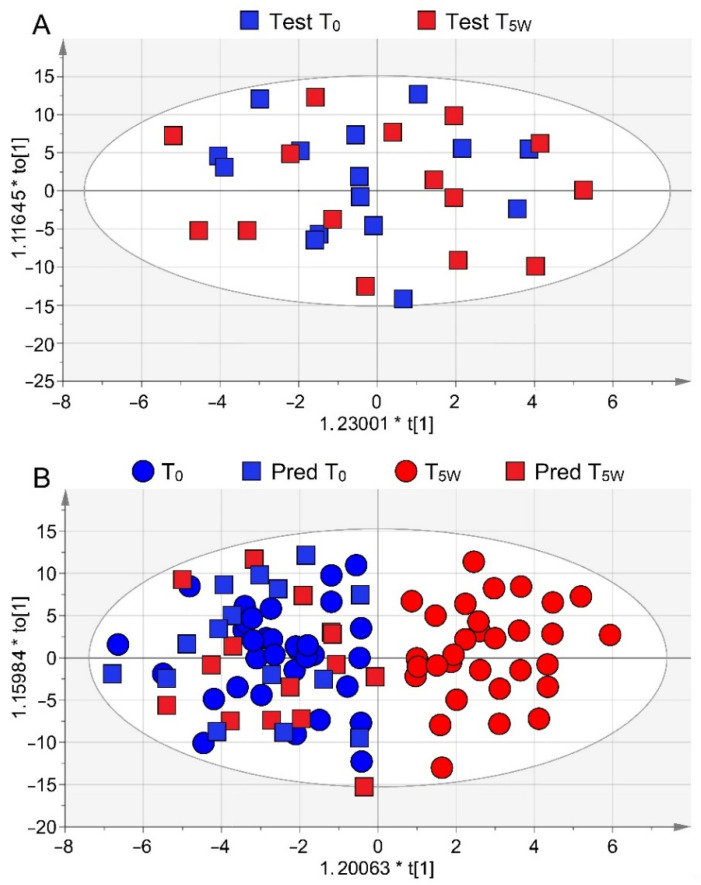
Validation of the T_0_–T_5W_ OPLS-DA model using a test set. (**A**) OPLS-DA model of the 15 patients not yet on PR. No satisfactory classification model was obtained (R^2^ = 0.12 and Q^2^ = 0.16), indicating that the two classes at T_0_ (Test T_0_, blue squares; Test T_5W_, red squares) and T_5W_ cannot be discriminated. (**B**) Predicted scores plot representing classification of the validation set obtained with samples projection onto the OPLS-DA T_0_–T_5W_ of Figure 2B. Circles represent the training set samples (T_0_, blue; T_5W_, red), while squares refer to the validation set samples (Pred T_0_, blue; Pred T_5W_, red). All predicted samples were correctly located at T_0_ since they were stable COPD patients not on PR.

The T_0_–T_5W_ model was validated by using the 15 COPD patients of the blind set. As expected, their EBCs collected at T_0_ and T_5W_ constituted a single class as they were not separated in the OPLS-DA scores plot (Figure 3A). Then, they were projected onto the statistical model of Figure 2B. This model (R^2^ = 0.79 and Q^2^ = 0.61, *p* = 0.001) correctly placed all T_0_ (blue squares) and T_5W_ (red squares) samples with those collected before starting PR (Figure 3B). This agrees with the fact that after 5 weeks no changes were present in the 15 stable non-rehabilitated patients (Table 1).

### 3.3. Correlation of Metabolomics with Clinical Data

**Figure 4:**
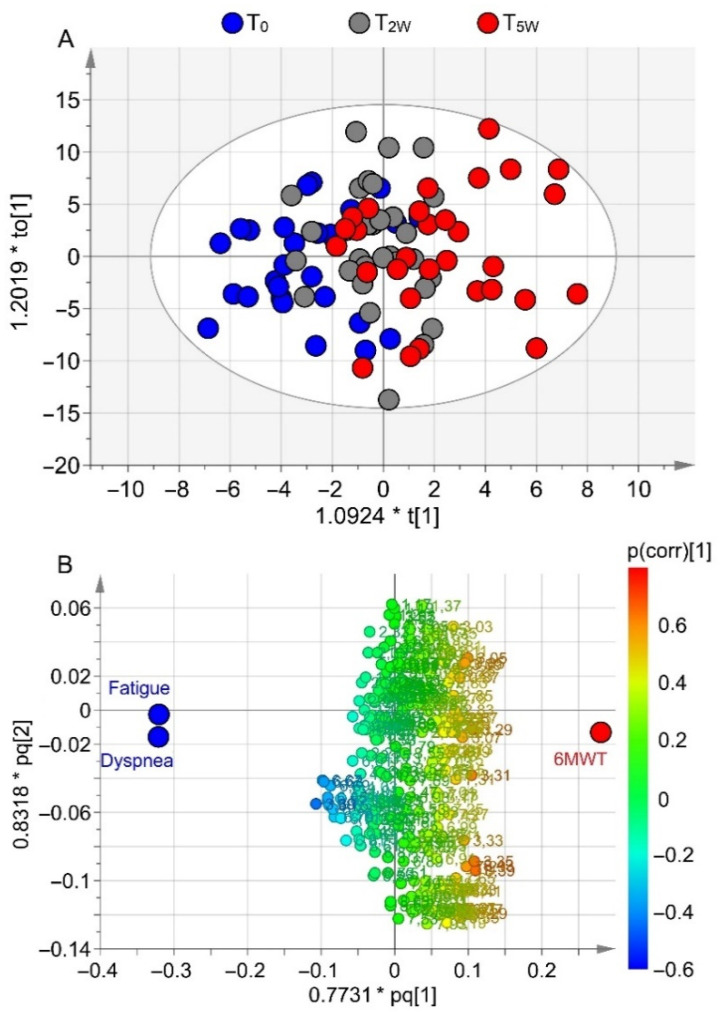
OPLS model including discriminating metabolites derived from NMR profiling of EBC and clinical outcomes evaluated during PR. (**A**) Scores plot showing the degree of separation among the classes T_0_ (blue circles), T_2W_ (gray circles), and T_5W_ (red circles). (**B**) Loadings plot associated with the OPLS analysis reported in (A), showing the parameters responsible for between-class separation. Numbers refer to buckets’ chemical shifts (spectral positions), while circles label high scores of dyspnea and fatigue (blue, at T_0_), and high values of 6MWT (red, at T_5W_). The pq[1] and pq[2] values refer to the weight that combines the X and Y loadings (p and q).

Figure 4A reports the model (R^2^ = 0.69, Q^2^ = 0.59, *p* = 0.009) obtained by combining NMR-derived metabolites and clinical variables at T_0_, T_2W_, and T_5W_. The three classes presented a progressive distribution along the predictive component (Figure 4A), reproducing the pattern of Figure 2A obtained for NMR parameters only. Furthermore, the T_0_ group (blue circles) is characterized by high scores of dyspnea and fatigue (blue circles in Figure 4B), and a low value of 6MWT. On the contrary, patients belonging to the T_5W_ class exhibited lower levels of fatigue and dyspnea and a longer distance in the 6MWT (red circle, Figure 4B). As in Figure 2A, the T_2W_ group overlapped with both T_0_ and T_5W_ classes. The variations observed for dyspnea, fatigue, and 6MWT in the OPLS model are reported in Figure S3. Since dyspnea and fatigue are discrete variables (i.e., they assume a finite number of isolated values), the plot presents an up-and/or-down distribution around steady values (Figure S3A,B), but the decreasing trend is well evident for both. On the contrary, the increasing 6MWT values show a more regular behavior (Figure S3C) because 6MWT is a continuous variable that can assume an infinite number of different values.

**Figure 5:**
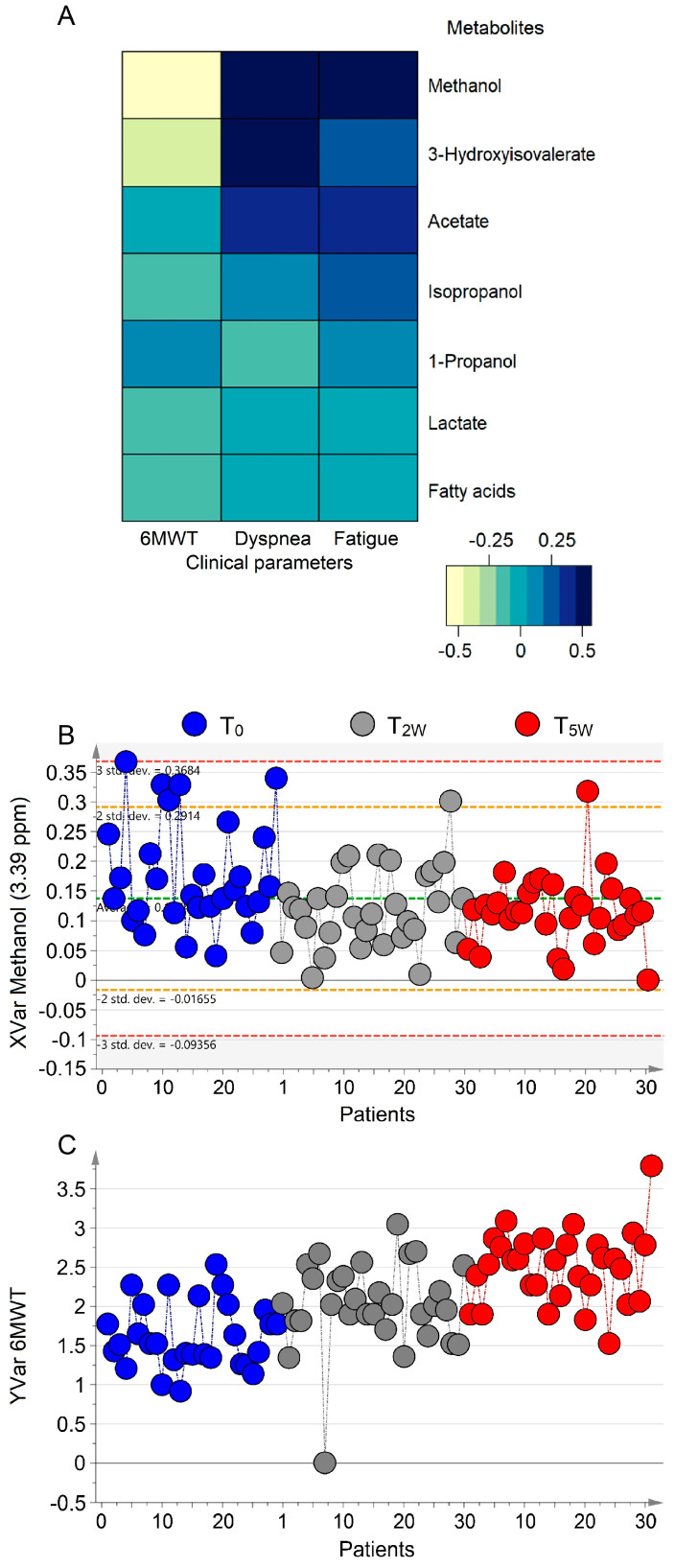
Correlation between molecular and clinical parameters. (**A**) Heatmap based on Pearson correlation coefficients between metabolites and clinical data. (**B**) Decrease of methanol values during the PR 5-week program at T_0_ (blue dots), T_2W_ (gray dots), and T_5W_ (red dots). (**C**) Corresponding increase of the distance in the 6MWT. In (**A**), blue tone indicates positive correlations between NMR and clinical parameters, whereas light tones indicate negative correlations. The strongest correlations are for methanol (see text). (**B**) XVar (methanol) and (**C**) Yvar (6MWT) show the values for the “molecular response” and distance variations during PR. Numbers on the *x*-axis refer to EBC samples of COPD patients involved in the study; the *y*-axis reports the variation.

The corresponding coefficient plots (Figure S4) express how strongly the clinical factors relate to the EBC metabolites. Among all, methanol showed the highest correlation: its levels are high in patients presenting high values of dyspnea and fatigue before starting activity at T_0_ (Figure S4A,B). After 5-week PR, methanol concentration decreased inversely with the lengths gained during the walk test (Figure S4C), and proportionally with the lower fatigue and dyspnea for each patient. Figure 5A reports the heatmap correlation matrix relating statistically discriminant metabolites with 6MWT, dyspnea, and fatigue parameters. The strongest correlation of methanol is represented by the dark-blue color, which indicates the positive correlation with dyspnea and fatigue, while the yellow-pale color represents the negative correlation with 6MWT. This indicates that molecular.

And clinical parameters are strictly related, as illustrated in Figure 5B,C, which depict the lowering concentration of methanol (panel 5B), and the “physical outcome”, expressed by the increase of the distance in 6MWT (panel 5C). Such a relationship strongly suggests that methanol can become a useful biomarker to monitor PR outcomes.

### 3.4. Metabolomics and Walk-Distance Paths

**Figure 6:**
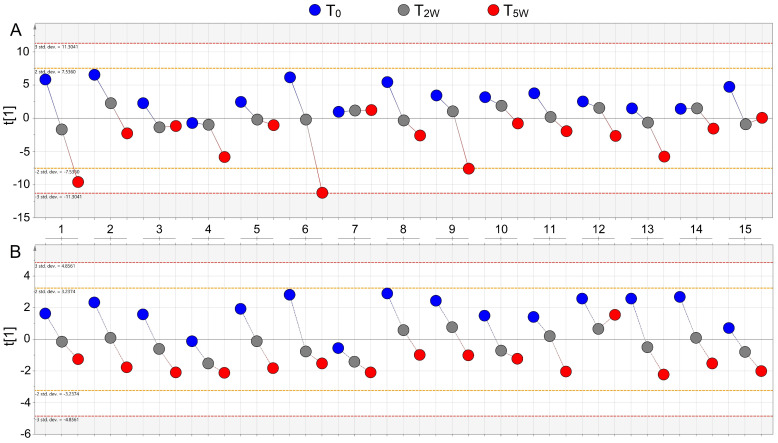
Comparison between metabolomic and distance patterns of 15 representative COPD patients during the whole T_0_–T_2W_–T_5W_ PR cycle. (**A**) Metabolomic path from the OPLS-DA model obtained with NMR parameters. (**B**) Walk path from the OPLS model considering the walked distances as y-variables. The numbers on the *x*-axis between the panels label the COPD patients.

Figure 6 compares the molecular parameters derived from the NMR profiles (panel A), and the model obtained using the walk distances (panel B) of 15 representative patients, during the PR cycle. For patients 1, 2, 5, 6, 8, 9, 10, 11, and 13 a linear evolution of the outcomes is observed in both analyses: the distance in the 6MWT increases linearly (panel B), with the corresponding linear changes in the molecular parameters (panel A).

According to the walked distances (panel B), patients 4, 7, 12, and 15 showed little improvement on going from T_0_ to T_5W_, with patient 12 showing a distance reduction from T_2W_ to T_5W_. Interestingly, their corresponding metabolite variations appear to be limited (T_0_–T_2W_ for patient 14; T_0_–T_2W_–T_5W_ for patient 7; and T_2W_–T_5W_ for patient 15). A different response was observed for patients 3 and 14 (T_2W_–T_5W_ metabolic changes being constant while the distance increased), and patient 12 (reduced T_2W_–T_5W_ distance and increased response in the metabolic changes) (Figure 6). The above data confirm that PR induces parallel alteration in the metabolic profile of and in clinical parameters, firmly linking molecular markers and PR outcomes.

## 4. Discussion

Our data indicate that the COPD metabotype evolves during PR. In particular, clear changes in dyspnea, fatigue, and 6MWT alter the metabotype during in-hospital PR. Among the metabolites, methanol decline in EBC well correlated with reduction of dyspnea and fatigue and increase of walk distance in the 6MWT, becoming a potential biomarker of COPD rehabilitation. Such a finding is of importance because methanol can become a tool to verify the effectiveness of PR in COPD patients. This is expected to remove the bias stemming from personal evaluation of the outcomes. In particular, patients will not be required to rate the evolution of the symptoms during PR or transform the severity of dyspnea and muscle fatigue into a numerical value. Possibly, a direct measurement of EBC methanol could indicate the adherence of a COPD patient to a specific PR program, and its assessment could eliminate all possible variables (personal response to a protocol, personal application, motivation, etc.). We are currently working on a methanol-based sensor for a portable instrument to be used in PR. Comparison between metabolic data and clinical parameters (Figure 6) suggests that PR and metabolic variations fuel each other, with positive clinical outcomes “requiring” a downgrading of metabolites worsening COPD.

Methanol occurs naturally in humans. It derives from the intestinal flora, fruit, vegetable, or alcoholic beverage consumption. Another endogenous source of methanol is via protein carboxymethylation (i.e., the methylation of amino acid COOH groups) that is catalyzed by methyltransferases or methyl esterase, which produces methanol (for a detailed review see [25]). The main way of elimination of methanol is oxidation. In humans, the oxidation of methanol (and ethanol) requires several stages of conversion; the most important one involves alcohol dehydrogenase (ADH) 1b, which catalyzes up to 90% of methanol (and ethanol) oxidation in the liver. Methanol is oxidized to formaldehyde, and formaldehyde to formic acid, which can either be excreted in the urine, or further oxidized to carbon dioxide. Formaldehyde, the main oxidation product of methanol, exacerbates airways inflammation in A549 alveolar and BEAS-2B bronchial cell lines [26], and in male Wistar rats [27]. In COPD, methanol levels increased with respect to asthma, with considerably reduced levels of formate [28], which is known to exert a protective role on lung cancer cell lines. The exhaled breath concentration of methanol is amplified in lung cancer, and COPD is characterized by an increased risk of lung carcinoma [29]. Therefore, methanol decline during PR might be related to an endogenous (direct or indirect) mechanism activated by PR to reduce pulmonary inflammation. As a consequence, the patients manifest an evident reduction of dyspnea and fatigue paralleled by an increased walk distance. This is in line with the observed improvements in skeletal muscle function and exercise capacity in COPD patients under PR [30], although no reduction of mediators of systemic inflammation was observed in urine. Most likely, “average” biological matrices like serum and urine reflect systemic rather respiratory inflammation [31], while the bronchoalveolar lavage (BAL) fluid and EBC appear to be a possible representation of lung metabolism with respect to plasma and urine [32,33].

This is the first report demonstrating that NMR metabolic profiling of EBC can be used to follow the PR outcomes of COPD patients, and that such profiling may be considered a fingerprinting of PR. Furthermore, the dynamical changes of EBC metabotype can be rapidly identified, and methanol is a potential biomarker of PR.

COPD is characterized by different phenotypes and clinical presentations [5]. Therefore, the principle “one size fits all” in PR [4] does not always yield the expected clinical outcomes as some individuals respond excellently, others discreetly, or do not respond at all [34,35,36]. Disease severity, the presence of comorbidities, motivation, as well as the number of sessions per week, the intensity and length of the treatment all affect the outcomes. Different time may be required by each patient, and failure to respond to a protocol indicates that it is not suitable for the patient while a different protocol could give the expected response. As stated [4], biomarkers should be one of the “future research directions” in PR: “Development of valid behavioral and physiological biomarkers that identify the suitability of a patient for a particular type of PR model. What factors determine which model best suits which type of patient? More evidence is urgently needed to help health professionals and patients make informed decisions on the basis of patient characteristics. This aligns with the emergence of personalized medicine”. Such a personalized strategy would favor the “right” delivery model and time for each patient and eliminate any bias in the evaluation of outcomes.

Our study, however, presents some limitations. First, although the number of patients exceeds that suggested by backward analysis, the sample size was relatively small. However, several measures were taken for quality control of the data. For EBC collection we minimized the external influence and contamination, all patients were well characterized according to current international guidelines, and differences among demographic parameters were all carefully minimized. A potential strength of our study was the presence of a validation cohort, since the external validation is the only discriminatory evidence that a calculated model can be clinically valuable, regardless of the reported predictive indices. For external validation, we decided to use a COPD cohort not yet on PR because a different group of PR patients could have introduced a bias related to different willingness to accomplish the cycle.

Second, since the patients involved were recruited through the hospital’s Pulmonary Rehabilitation Division, our selection adhered to the PR protocol, which excludes patients with severe comorbidities. Therefore, our metabolomics analysis awaits validation in patients with different degrees of comorbidity.

Third, lack of randomization between PR and control groups could also be considered a limitation. However, randomization in PR “violates the principle of clinical equipoise” [37]; therefore, it is not ethical because patients are either in or out of the PR program, and when started, each patient has to finish the cycle. We could have established a different PR regime for selected patients, but, again, this is ethically controversial [37].

Notwithstanding the above limitations, we were able to recognize specific differences in the metabolomic patterns of COPD along the PR cycle. Furthermore, a clear correlation was found between the metabolic response of patients and the clinical outcomes, being the reduction of methanol related to the reduction of dyspnea and fatigue, and the increased walk distance. As such, EBC methanol concentration could become a molecular tool to measure the PR effectiveness avoiding all possible bias related to personal feeling of the patients. A step forward would be the identification of the molecular threshold of methanol that typifies the metabotype changes during COPD rehabilitation. However, since the molecular evolution between contiguous pathophysiological states is not “linear” because of the presence of overlapping “molecular zones”, an increased population of patients should be studied. In addition, a longer PR period should be considered and, because the beneficial effects of rehabilitation treatment tend to fade over time [38], a control on the persistence of the effects would be needed. Unfortunately, the regional admittance regulation indicates a maximum of 5-week hospitalization for PR, and among the patients continuing physical rehabilitating activity personally, a very low number was considered trustworthy.

All considered, the reported results provide sufficient evidence on the application of metabolomics to PR, opening new routes for metabolomics-guided management of rehabilitation. Furthermore, correlation of molecular data with clinical parameters suggests that even a single biofluid from the lung compartment can generate a reasonable understanding of a complex system, which is totally described by the clinical parameters. We also showed that COPD patients undergoing PR present changing phenotype at different time of the treatment, and those changes can strongly suggest the “right” protocol and time for the treatment.

## 5. Conclusions

This study showed that NMR-based metabolomics of EBC may be used to monitor PR in COPD patients. It is basically noninvasive and provides quite real-time answers, particularly during the rehabilitation time ranges, offering an unbiased personalized approach with an optimal use of health care resources. We also showed that this approach is able to define the molecular evolution of COPD phenotype under PR, identifying specific biomarkers. In particular, methanol, being correlated with clinical parameters, can be used to monitor PR outcomes, therefore suggesting personalized protocols. Most likely, metabolomics can be extended to rehabilitation of other chronic diseases.

## Figures and Tables

**Table 1:** Clinical characteristics of COPD patients under PR and controls at the baseline (T_0_) and after 5 weeks (T_5W_).

Rehabilitation (*n* = 35)					Controls (*n* = 15)			
	1	2		3	4			
	T_0_	T_5W_	*p* Value (1–2)	T_0_	T_5W_	*p* Value (1–3)	*p* Value (2–4)	*p* Value (3–4)
**Age (year)**	70.7 ± 7.7	-	-	70.7 ± 5.7	-	0.90	-	-
**Sex (M/F)**	31/4	-	-	15/0	-	0.82	-	-
**BMI (kg/m^2^)**	29.8 ± 6.04	-	-	26.75 ± 2.28	-	0.060	-	-
**Smoke**	31 × 10^−4^ S	-	-	24 × 10^−2^ S	-	0.99	-	-
**GOLD C (n)**	2	-	-	1	-	0.99	-	-
**GOLD D (n)**	33	-	-	14	-	0.99	-	-
**FEV_1_ (L)**	1.19 ± 0.40	1.24 ± 0.43	0.29	1.22 ± 0.39	1.21 ± 0.31	0.80	0.81	0.61
**FEV_1_ (%)**	48.03 ± 13.66	50.35 ± 15.39	0.21	42.27 ± 8.63	42.17 ± 7.53	0.12	0.056	0.63
**FVC (L)**	2.59 ± 0.72	2.50 ± 0.80	0.56	2.48 ± 0.52	2.47 ± 0.45	0.56	0.030	0.82
**FVC (%)**	80.57 ± 16.14	78.44 ± 20.51	0.37	68.67 ± 11.24	69.17 ± 10.24	0.010	0.073	0.52
**FEV_1_/FVC**	47.4 ± 15.45	50.68 ± 13. 96	0.01	47.40 ± 10.26	47.55 ± 10.19	0.99	0.44	0.66
**FEF_25-75_ (L/s)**	1.99 ± 1.68	2.14 ± 1.6	0.04	1.08 ± 0.52	1.18 ± 0.51	0.040	0.028	0.21
**SaO_2_ (%)**	92.51 ± 5.06	93.42 ± 5.12	0.03	91.52 ± 5.41	92.12 ± 5.01	0.80	0.41	0.45
**6MWT (m)**	191.11 ± 50.26	298.54 ± 66.74	<0.001	211.23 ± 48.36	210.24 ± 55.71	0.31	<0.0001	0.62
**Fatigue**	8.31 ± 2.23	2.71 ± 2.08	<0.001	7.81 ± 2.13	7.73 ± 2.18	0.52	<0.0001	0.34
**Dyspnea**	9.06 ± 1.61	2.86 ± 1.97	<0.001	8.76 ± 1.82	8.73 ± 1.57	0.36	<0.0001	0.71

M, males; F, females; BMI, body mass index; GOLD, global obstructive lung diseases classification; FEV_1_, forced expiratory volume in 1 s; FVC, forced vital capacity; FEF_25-75_, forced expiratory flow; SaO_2_, oxygen saturation; 6MWT, 6 min walk test (in meters, m).

**Table 2:** Misclassification table of the EBC samples from COPD patients under PR according to the OPLS-DA model of Figure 2. The gray diagonal highlights the T_0_, T_2W_, and T_5W_ patients that are correctly classified: 27/31 at T_0_, 9/31 at T_2W_, and 25/31 at T_5W_, and the unclassified patients.

	T_0_	T_2W_	T_5W_	Members	Correct (%)
**T_0_**	27	2	0	29	93.10
**T_2W_**	12	9	10	31	29.03
**T_5W_**	0	6	25	31	80.65
**No Class**	0	0	0	0	
**Total**	39	17	35	91	67.03
**Fisher’s Prob.**				6.7×10^−7^	

## Data Availability

The data presented in this study are available in the article and supplementary material.

## Supplementary Materials

The following are available online at https://www.mdpi.com/article/10.3390/cells11030344/s1, Table S1: Statistically significant discriminating metabolites in the T0–T5W OPLS-DA model; Figure S1: Box-and-whisker plots showing the concentration levels of discriminating metabolites for the T0–T2W–T5W and T0–T5W OPLS-DA models; Figure S2: Box-and-whisker plots showing the concentration levels of discriminating metabolites for the T0–T5W OPLS-DA model; Figure S3: Plots of the YVar values for the three clinical parameters obtained from the OPLS model correlating NMR parameters and clinical outcomes; Figure S4: Coefficient plots correlating metabolites uncovered in the NMR profile of EBC samples and the clinical rehabilitation parameters.
