# Supplementary material for: Metabolomics of COPD Pulmonary Rehabilitation Outcomes via Exhaled Breath Condensate
Source: Cells. 2022 Jan 20;11(3):344. doi: 10.3390/cells11030344 (PMC8834302; doi:10.3390/cells11030344)
Supplement: Supplementary file 1 [file cells-11-00344-s001.zip › cells-1528661-supplementary.pdf]

Supplementary material

Metabolomics of COPD Pulmonary Rehabilitation Outcomes via Exhaled Breath Condensate

Mauro Maniscalco [1,†,*], Debora Paris [2,†], Paola Cuomo [2], Salvatore Fuschillo [1], Pasquale Ambrosino [3], Annabella Tramice [2], Letizia Palomba [4] and Andrea Motta [2,*]

[1] Istituti Clinici Scientifici Maugeri IRCCS, Pulmonary Rehabilitation Unit of Telese Terme Institute, 82037 Telese Terme, Italy; salvatore.fuschillo@icsmaugeri.it
[2] Institute of Biomolecular Chemistry, National Research Council, 80078 Pozzuoli, Italy; debora.paris@icb.cnr.it (D.P.); pao.cuomo@gmail.com (P.C.); annabella.tramice@icb.cnr.it (A.T.)
[3] Istituti Clinici Scientifici Maugeri IRCCS, Cardiac Rehabilitation Unit of Telese Terme Institute, 82037 Telese Terme, Italy; pasquale.ambrosino@icsmaugeri.it
[4] Department of Biomolecular Sciences, University of Urbino "Carlo Bo", 61029 Urbino, Italy; letizia.palomba@uniurb.it
* Correspondence: mauro.maniscalco@icsmaugeri.it (M.M.); andrea.motta@icb.cnr.it (A.M.)
† These authors contributed equally to this work.

Table S1. Statistically significant discriminating metabolites in the T_0-T_{5W} OPLS-DA model of Figure 2A. The arrows indicate a decrease (↓) or an increase (↑) of the metabolite.

Metabolites	Chemical shift (δ, ppm)
Acetate ↓	1.93
Methanol ↓	3.39
3-Hydroxyisovalerate ↓	1.25; 2.37
Isobutyrate ↓	2.39
Acetone ↓	2.23
Isopropanol ↑	4.03
1-Propanol ↑	3.56
Lactate ↑	1.33
Fatty acids ↑	1.30

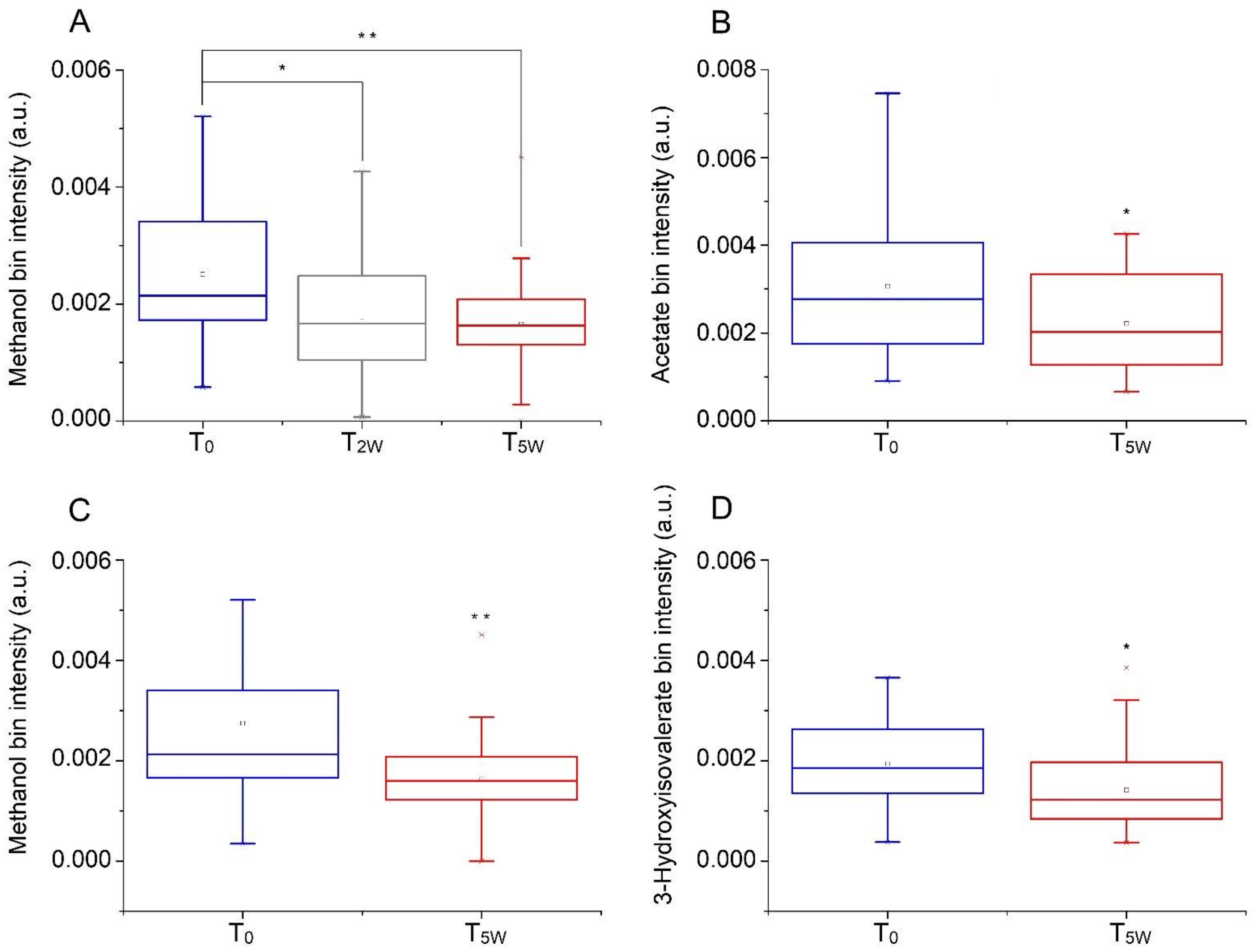

Figure S1. Box-and-whisker plots showing the concentration levels of discriminating metabolites in the T_0-T_{2W}-T_{5W} and T_0-T_{5W} OPLS-DA models. (A) methanol at T_0, T_{2W} and T_{5W}, (B) acetate at T_0 and T_{5W}, (C) methanol at T_0 and T_{5W}, and (D) 3-hydroxyisovalerate at T_0 and T_{5W}. Boxes show median (horizontal line in each box), mean (the empty box), 25th and 75th percentiles (edges of box), maximum and minimum values (whiskers), and the outliers (cross). ANOVA test significance is reported as *, p<0.05 and **, p<0.001.

.

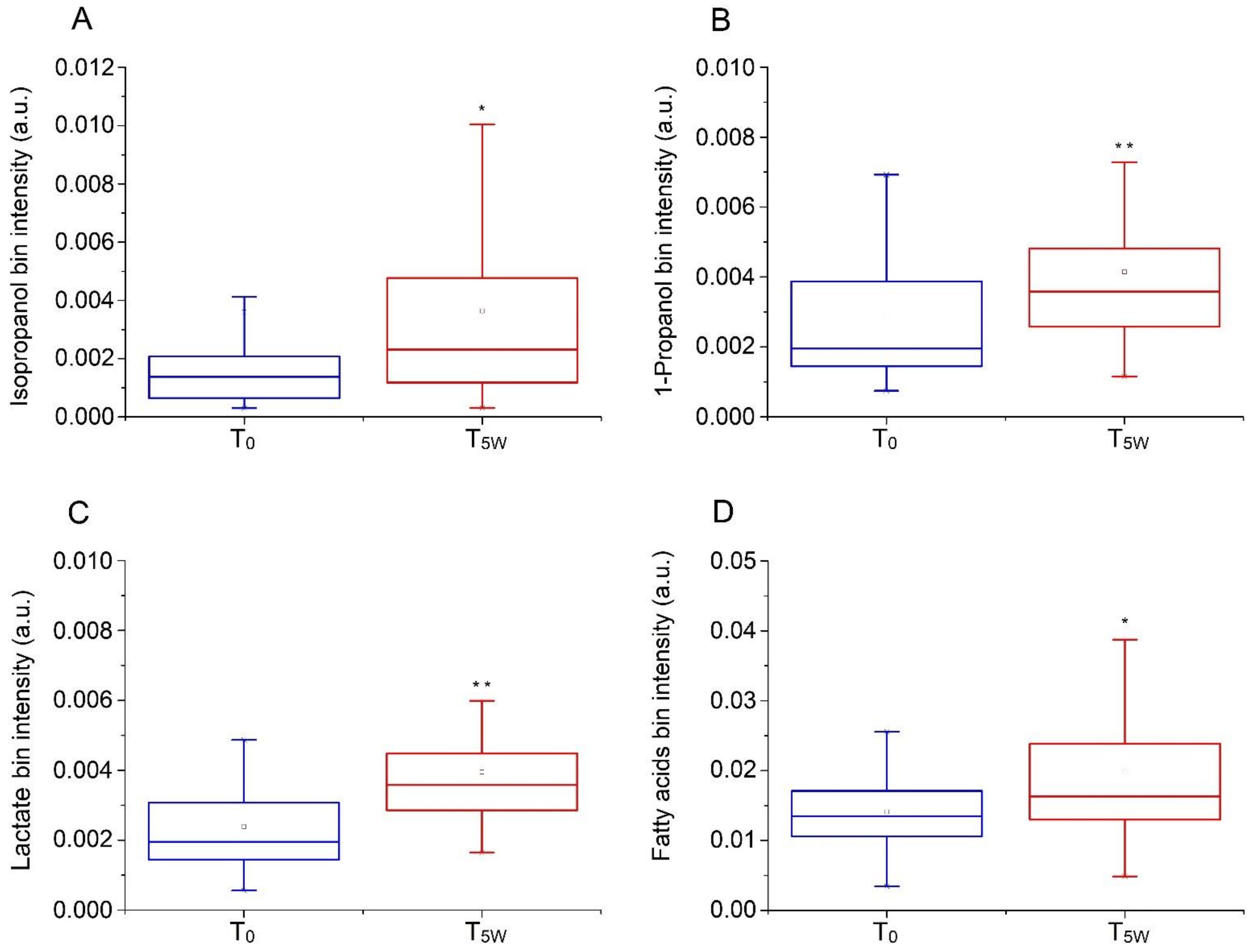

Figure S2. Box-and-whisker plots showing the concentration levels of discriminating metabolites in the T_0-T_{5W} OPLS-DA model. (A) isopropanol, (B) 1-propanol, (C) lactate, and (D) fatty acids. Boxes show median (horizontal line in each box), mean (the empty box), 25th and 75th percentiles (edges of box), maximum and minimum values (whiskers), and the outliers (cross). ANOVA test significance is reported as *, *p*<0.05 and **, *p*<0.001.

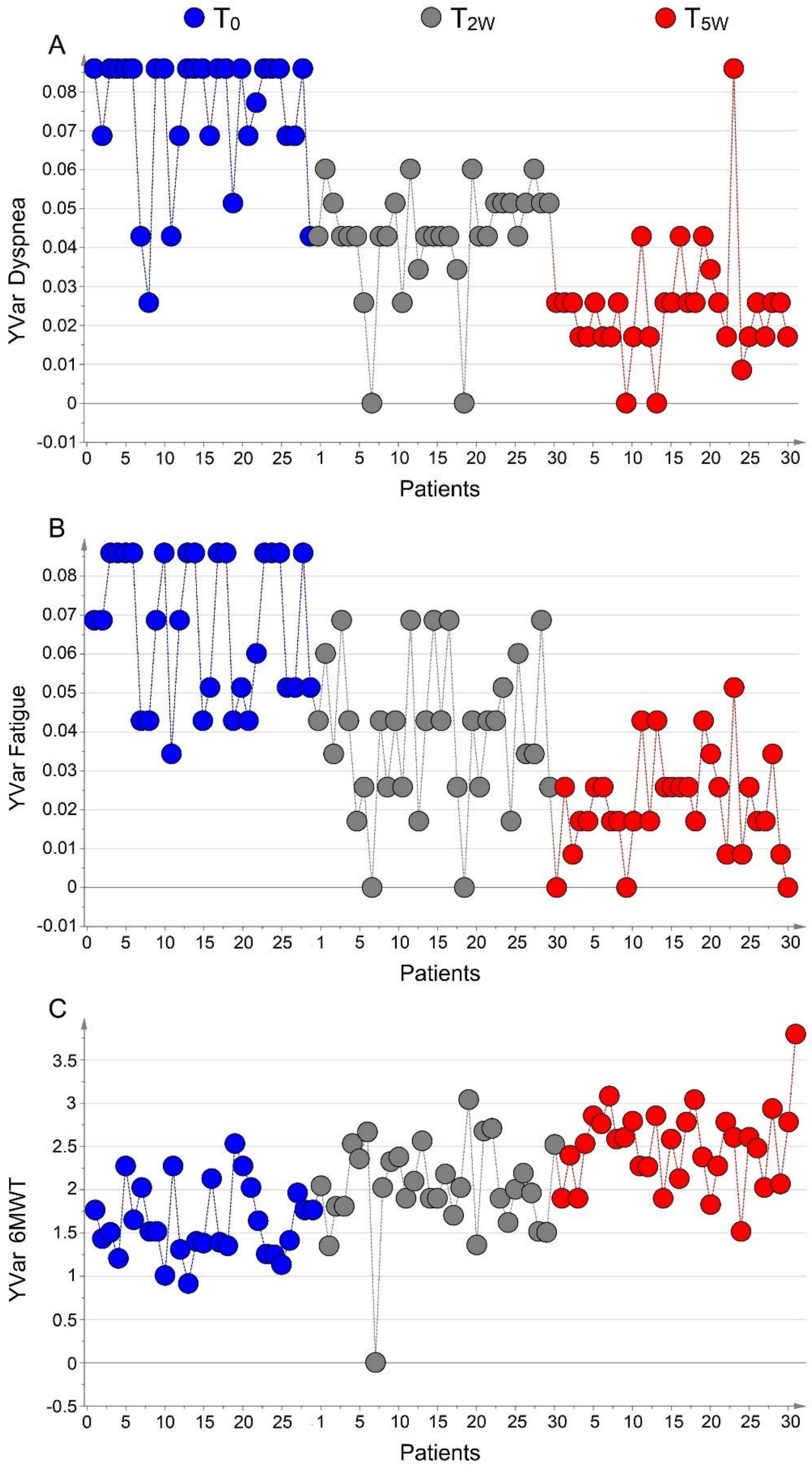

Figure S3. Plots of the YVar values for the three clinical parameters obtained from the OPLS model correlating NMR parameters and clinical outcomes. Variations of (A) dyspnea, (B) fatigue, and (C) 6MWT at T_0 (blue dots), T_{2W} (gray dots), and T_{5W} (red dots). Numbers on the x-axis refer to EBC samples of COPD patients involved in the study; the y-axis reports the variation (see main text).

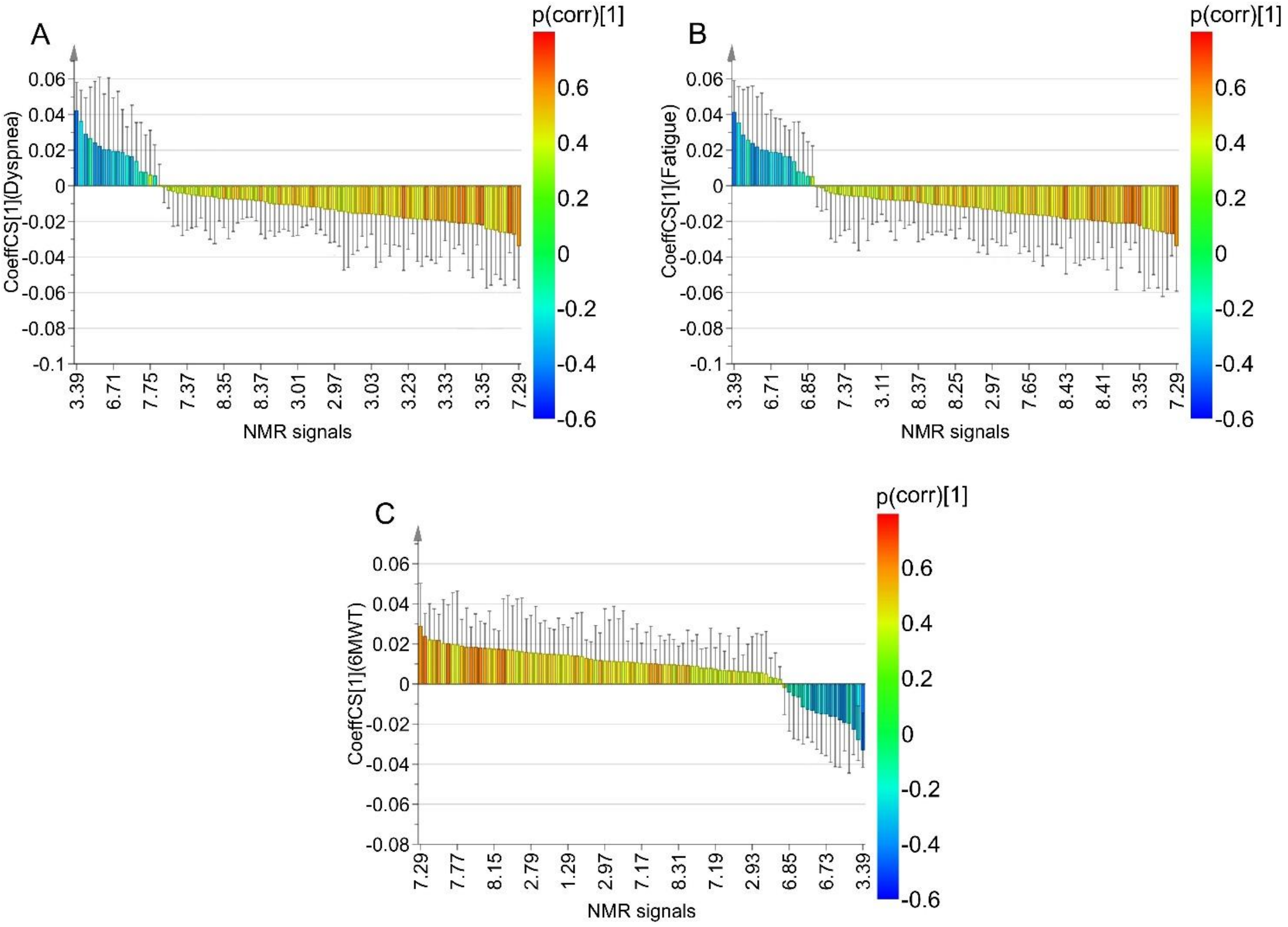

Figure S4. Coefficient plots correlating metabolites uncovered in the NMR profile of EBC samples and the clinical rehabilitation parameters. Among all, methanol (3.39 ppm) showed the highest correlation with dyspnea (A), fatigue (B) and 6MWT (C), presenting high values of fatigue and dyspnea at T_0. After 5 weeks of rehabilitation, methanol concentration decreased inversely with the lengths gained during the walk test, and proportionally with the low measures of fatigue and dyspnea reported for each patient.
